# The impact of time pressure and type of fraud on susceptibility to online fraud

**DOI:** 10.3389/fpsyg.2025.1508363

**Published:** 2025-04-10

**Authors:** Ce Lyu, Shenghan Gao, Qingqi Zhang

**Affiliations:** ^1^School of Sociology, China University of Political Science and Law, Beijing, China; ^2^School of Criminal Justice, China University of Political Science and Law, Beijing, China

**Keywords:** online fraud, time pressure, type of fraud, susceptibility to fraud, Internet fraud

## Abstract

With the rapid development of the Internet, online fraud has evolved, posing a serious threat to people’s financial security. Susceptibility to online fraud refers to an individual’s vulnerability to Internet fraud, which was assessed in this study using the fraud material assessment paradigm. This study employed a 2 × 2 two-factor mixed experimental design to examine the effects of time pressure (present vs. absent) and fraud type (profit-taking vs. loss-avoidance) on susceptibility to online fraud. In the pre-study, real-life Internet fraud cases were adapted into legitimate and illegitimate materials, which were then rated. In the formal experiment, participants in the pressure group and control group assessed the legitimacy of these materials. The findings revealed that: (1) time pressure had a significant main effect, with participants exhibiting higher susceptibility to online fraud under time pressure than without it; (2) fraud type had a significant main effect, with susceptibility being higher for loss-avoidance fraud compared to profit-taking fraud; (3) a significant interaction effect was observed, where time pressure increased susceptibility to loss-avoidance fraud but had no significant effect on profit-taking fraud. These results suggest that time constraints and fraud types interact to influence an individual’s ability to resist online fraud. The findings provide insights for fraud prevention strategies, emphasizing the need to mitigate time pressure effects and educate individuals on different fraud types.

## 1 Introduction

In recent years, with the further popularization and development of Internet technology, the functions carried by cyberspace tend to be diversified. People’s daily needs, such as shopping, food delivery, transportation, health care and financial services, have become highly integrated into internet platforms, with online payment and money transfers emerging as indispensable components of modern activities. As of June 2023, the number of Internet users in China reached 1.079 billion, and the Internet penetration rate reached 74.4% (China Internet Network Information Center, 2023). On the one hand, the popularity of the Internet has helped China to quickly realize informatization, on the other hand, it has also provided a hotbed for the rapid development of a new form of fraud—online fraud, as defined in Article 2 of the Anti-Telecommunications and Network Fraud Law of the People’s Republic of China (2019), refers to the act of fraudulently obtaining public or private property by means of telecommunications or network technology, through remote or non-contact methods, with the intention of illegal possession. Meanwhile, online fraud does not occur in isolation but often intersects with other financial crimes such as money laundering and corruption. For instance, scammers may use fraudulent schemes to obtain illicit funds, which are then laundered through complex financial transactions. The attempts to control online fraud has always been a struggle, although China has achieved certain results in cracking down on online fraud, the number of this type of crimes remains high. According to data released by the Ministry of Public Security, from January to November 2023, the Ministry of Public Security detected more than 391,000 online fraud cases, involving significant economic loss: Though in 2019, the number of online fraud cases decreased by 3.1% year-on-year, and the number of cases filed decreased by 17.3% year-on-year, the amount involved only decreased by 1.3% year-on-year. It can be seen that although considerable results have been achieved in anti-fraud work, the endless stream of new frauds by fraudmers still make many people prey. From January to October 2020 alone, more than 260 new types of frauds appeared, posing a great threat to people’s property safety. On a global scale, one report estimated that fraud costs the world economy over $5 trillion per year, highlighting the widespread and persistent nature of fraudulent activities (Gee and Button, 2019). In addition to the loss of property, the experience of being cheated can also bring serious psychological trauma and may even induce the victim to commit suicide (Luo, 2017). Also, a survey by the European Commission (2020) showed that 79% of scam victims have suffered emotionally, whereas only 24% have suffered financially. As online fraud has become one of the key objects of crime control in China, this thesis attempts to find out the factors that affect individual’s susceptibility to fraud through experiments, and provide theoretical support for public security education against online fraud.

In criminology, “susceptibility” is defined as the likelihood of a person becoming a victim (Skidmore et al., 2020). However, from the perspective of psychology, “susceptibility to online fraud” refers to the degree of vulnerability of individuals to online fraud (Gao, 2021). Currently, there are three primary methods for measuring fraud susceptibility: deceived experience reporting, scale testing, and susceptibility testing. Each method has distinct strengths and limitations.

To value which method properly matches the aim of the study, starting from the deceived experience reporting method, which relies on participants self-reporting whether they have been deceived. This approach is widely used in deep learning modeling and large-scale surveys due to its ease of implementation. However, it is primarily suitable for qualitative research, as it lacks experimental control and may be influenced by memory biases or underreporting.

Scale testing has traditionally focused on measuring fraud susceptibility through victims’ understanding of fraud (Harrison et al., 2016) or their sensitivity to persuasion (Susceptibility to Persuasion, StP) (Modic et al., 2018; Modic and Lea, 2013). While scale testing is convenient for large-scale data collection and useful for examining relationships between variables, it suffers from a major limitation: it cannot impose strict experimental controls, making it less suitable for studying causal mechanisms in experimental settings. Furthermore, existing fraud susceptibility scales are limited in number and may not comprehensively capture susceptibility across different fraud types.

Susceptibility testing includes network attack testing and fraud material evaluation (Gao, 2021). In network attack testing, researchers simulate real cyberattacks on consenting participants and analyze their responses (Harrison et al., 2016; Harrison et al., 2015; Vishwanath et al., 2011; Moody et al., 2017). This method has high external validity because it closely mimics real-world fraud encounters. However, it is primarily focused on phishing emails in foreign research contexts, which may not fully reflect the diverse and fast-evolving fraud landscape in China (Wang et al., 2012; Luo et al., 2013). Additionally, ethical concerns regarding cybersecurity risks limit its applicability.

By contrast, fraud material evaluation involves presenting fraudulent materials (e.g., phishing emails, lottery scams) to participants and assessing their ability to identify them as fraudulent. This method avoids security and ethical concerns, offers greater experimental control over environmental variables, and allows researchers to adapt materials based on emerging fraud trends, making it more timely and practical than network attack testing. Considering these factors, this study selected the fraud material evaluation method as the most suitable approach. This method allows for better control of experimental conditions, particularly time pressure, and ensures that the susceptibility measurement remains relevant to evolving fraud tactics.

Current research on fraud susceptibility can be broadly categorized into three main areas: (a) the nature of fraud, (b) demographic characteristics and susceptibility to frauds, and (c) individual differences (Hanoch and Wood, 2021; Jones et al., 2015; Jones et al., 2019). Empirical studies have predominantly focused on the latter two, aiming to better predict who is more vulnerable to fraud. However, a significant gap exists in the quantitative empirical research on the nature of fraud. Therefore, this study seeks to address this gap by employing controlled, manipulable experimental settings to further investigate the psychological mechanisms underlying susceptibility to fraud. A deeper understanding of why individuals fall victim to fraud can ultimately contribute to the development of more effective preventive measures. Drawing on prior studies and the potential for laboratory control, we selected time pressure and fraud type as the key manipulated variables.

Time pressure refers to the individual’s feeling of stress caused by time constraints in the process of tasks. In previous studies, time pressure is one of the important influencing factors of fraud susceptibility. Wang et al. found through experiments that time constraints in fraudulent messages increase the probability of college students’ response behavior (Wang et al., 2012). Other researchers have constructed heuristic-systematic models and found through experiments that time pressure has a significant positive impact on the probability of college students being deceived (Luo et al., 2013). When the victim is involved in fraud, the fraudmers often induces the victim to feel anxious and sleepy by applying time pressure and other methods, resulting in cognitive bias and emotional imbalance (Norris and Brookes, 2021), thus enhancing the victim’s susceptibility to fraud. Harrison et al. found that message arguments in phishing emails tend to be brief and often rely on urgent prompts, using words such as “warning” or “deadline, “and phrases suggesting loss, such as “The account is about to closed” (Harrison et al., 2015). The fraudmers intend to make these phrases elicit an “emotional response” and lead the recipient to act quickly, bypassing their more rational core decision-making process and ignoring other clues that may highlight the illegitimacy of the message (Vishwanath et al., 2011).

Based on this, this study puts forward Hypothesis 1: The main effect of time pressure is significant, and the subjects are more susceptible to online fraud under the condition of time pressure.

Time pressure does not work alone in frauds, but is often combined with other factors (or affects other factors, such as state emotions, cognitive resources, etc.), thus affecting individuals’ susceptibility to fraud. Chen Hongmin et al. believed that frauds mainly include two types. One is to forge “accidental acquisition,” defraud the victim’s initial trust by creating a situation of “profit,” and lead to decision-making deviation. The other is to fabricate “unexpected events” to defraud the victim’s distrust by creating a “loss” situation, and lead to decision-making bias (Chen et al., 2023). In this study, the former is defined as profit-seeking fraud and the latter as loss-avoiding fraud. In the face of losses and gains, individuals have different decision-making biases: prospect theory puts forward that people are risk-averse to gains and risk-seeking to losses (Kahneman and Tversky, 1979). For this result, Kahneman et al. mainly used the expectation theory to explain: the decision maker first simplifies the information into gains or losses, and then calculates the weight of mathematical functions according to the loss and gain information to achieve a decision that maximizes the effect. Creating loss situations with emotional phrases that incorporate elements of threat or fear is one of the common tactics used by fraudsters (e.g., suggesting that a bank account is about to be closed or suggesting that a bank account has been compromised). A content analytics study shows that fraudmers use this fear-inducing tactic in more than 60% of phishing emails (Kim and Kim, 2013). In this case, such fear-inducing content in fraud messages is often referred to as a “fear appeal” (Petty and Briñol, 2015) and may lead to increased acceptance of deceptive messages due to specific effects on information processing. Fear appeal is one of the most studied methods of provoking attitude change in the broader persuasion literature.

Cacioppo et al. (1997) the research of Cacioppo et al. on fear appeals and framing effects for more than 30 years shows that negative and fear-inciting information (i.e., threats, warnings and deadlines) is more prominent than positive and reward-based information in the process of information processing (Wegener and Petty, 2001). This finding is mainly supported by the research on negative bias, that is, individuals are more sensitive to negative information. Negative stimuli were preferentially detected at lower exposure levels compared to positive stimuli and elicited stronger or faster responses than positive events (Aarts and Dijksterhuis, 2003). Taken together, the negative bias suggests that fear-based fraud messages are more likely to cause victimization than positive or reward-based messages, mainly because fear appeals reduce the amount of attention an individual is likely to pay when receiving a fraud.

Based on this, this study puts forward Hypothesis 2: the main effect of fraud type is significant, and compared with profit-seeking frauds, the subjects under loss-avoiding frauds are more susceptible to online fraud.

With the deepening of research, the explanation of expectation theory shows certain limitations. Hu Weiguo studied the influence on time pressure and profit and loss framework in risk decision-making, and the results show that time pressure weakens the framework effect (Hu and Hu, 2009). Ben Zur and Breznitz (1981) proposed that time pressure has the potential to reverse the framework of perceived benefits, leading to an increase in risk aversion and adopting strategies that consume fewer cognitive resources, i.e., reduce appetite for risk (Ordóñez and Benson, 1997). Therefore, some scholars have proposed the Equate-to-differentiate model to explain the results of prospect theory. This model holds that individuals’ cognitive resources and rationality are limited, and making choices does not examine all dimensions completely according to the maximum benefit, but compares some dimensions and then makes choices (Li, 1994; Li, 2001). In short, human decision-making behavior is the process of subjectively searching for a certain option to be superior to another in some dimensions.

Moreover, this model holds that the framing effect works by affecting individuals’ perception of differences between different dimensions (best outcome and worst outcome) (Li, 2005): when Scheme A is better than Scheme B in the best case, and Scheme B is better than Scheme A in the worst case, two different judgment dimensions are produced: best outcome and worst outcome. At this time, the rationality of the decision-maker cannot support the comprehensive consideration of the maximum benefit of the whole under the two possible results. Only the one with the larger difference among the two dimensions can be selected for judgment, and the other dimension with the smaller difference is “harmonized” and does not participate in the decision-making consideration. In the loss situation, individuals are more sensitive to the difference perception of the best result, and tend to make judgments in the best situation dimension, so choose Scheme A, which is more advantageous in this dimension; In the income situation, individuals are more sensitive to the difference perception of the worst outcome, and tend to make judgments in the worst outcome dimension, so they choose Scheme B (Liu and Sun, 2014), which has more advantages in this dimension. At the same time, time pressure has an impact on the subjects’ cognitive strategies, so the role of profit and loss framework under time pressure needs further study.

In previous studies on the interaction between time pressure and fraud types, Hu Weiguo claimed the influence on time pressure and profit and loss framework in risk decision-making shows that time pressure weakens the framework effect. However, this study does not use the classic paradigm of manufacturing framework effect, but takes fraud type as a variable. At present, there is no conclusion about the interaction between time pressure and fraud type on fraud susceptibility. Grazioli’s (2004) research found that individuals who succeeded in identifying fraudulent websites noticed fewer cheating clues than individuals who did not. This counterintuitive behavior is interpreted as: when a strong clue of deception is found, the subjects stop searching. This is consistent with The Theory of Deception (Johnson et al., 1992; Johnson et al., 1993; Johnson et al., 2001), which suggests that the ability to detect deception lies not in a deeper search, but in the intensity of the fraud clue. According to the theory of deception, identifying inconsistent cues (e.g., too-good-to-be-true guarantees, exaggerated claims of product merits) is necessary for successful detection (Grazioli, 2004). In profit-seeking frauds, “gaining benefits” is a strong inconsistent deceptive clue, so the subjects immediately make a “fraudulent” judgment. However, loss-avoiding frauds do not have the “strong fraud clues” in profit-seeking frauds. Individuals need longer cognitive processing time and higher cognitive resources for loss-avoiding frauds, which requires more systematic processing. However, compared with positive stimuli, negative stimuli are detected first at lower exposure levels and cause stronger or faster responses than positive events (Aarts and Dijksterhuis, 2003). Therefore, individuals in the situation of negative stimuli loss-avoiding frauds tend to react immediately instead of carrying out fine systematic processing. At the same time, under the influence of time pressure, it is more difficult for the subjects to carry out systematic cognitive processing (Luo et al., 2013), which is not conducive to the subjects’ identification and evaluation of fraud clues. The theory of limited cognitive resources also points out that based on the limitation of cognitive resources, individuals’ attention to information in the process of interpersonal interaction is selective (Lindenberg, 2001). In a specific situation, individuals can only pay attention to part of information, while others are in a state of attention overflow, which becomes the background of behavior, and the information selected by attention has a greater influence on individuals’ trust decision in the process of interpersonal interaction. In profit-seeking frauds, strong fraud clues make them the information chosen by attention, thus affecting the judgment of subjects in trust decision-making. Even under time pressure, this strong clue can still be well captured by subjects as the strong fraud clues chosen by attention, so that subjects can correctly identify the fraud situation. However, for loss-avoiding frauds, time pressure will significantly reduce the individual’s attention to the fraud clues, thus failing to distinguish the fraud situation, and enhance the individual’s susceptibility to online fraud.

Based on this, this study puts forward Hypothesis 3: There is an interaction between time pressure and fraud type. Compared with profit-seeking frauds, the susceptibility of individuals who experience loss-avoiding frauds to online fraud is more affected by time pressure.

## 2 Materials and methods

### 2.1 Materials

To enhance the authenticity and ecological validity of the experiment, this study adapts real-world fraud cases into experiment materials and evaluates their effectiveness. The goal is to develop simulated fraud scenarios that can be used in future research while ensuring proper manipulation of the independent variables. Specifically, the materials are categorized into profit-seeking and loss-avoiding scenarios, further divided into legal and illegal contexts.

#### 2.1.1 Material compilation

Chen Hongmin et al. believed that frauds mainly include two types, one is forging “accidental gains” and the other is fabricating “accidental events” (Chen et al., 2023). This study defines the former as profit-seeking frauds and the latter as loss-avoiding frauds. Drawing lessons from the existing methods of compiling fraudulent advertising stimulus materials in previous studies (Gao, 2021), and combining with a variety of real fraud situations that have appeared in online social platforms in recent years, profit-seeking and loss-avoiding situation materials are compiled according to the type of fraud, with 4 in each group.

Refer to Luo’s (2022) method of compiling fraud and real investment project materials, the legal situation materials of this study choose real situations in life, such as obtaining SMS verification codes, receiving advertising emails from official website, etc. According to the nature of the content, it is compiled into profit-seeking and loss-avoiding situational materials, with 4 in each group. The material types are shown in Table 1.

**Table 1:** Classification of fraud scenario materials.

	Profit-seeking	Loss-avoiding
Legal	Legal × Profit-seeking	Legal × Loss-avoiding
Illegal	Ilegal × Profit-seeking illegal	Illegal × Loss-avoiding

#### 2.1.2 Material assessment

A total of 18 participants with a certain psychological foundation were recruited to evaluate the simulated fraud situational materials compiled. Referring to the evaluation items of the previous textual materials, the legality, authenticity, logic of the text, content rationality, and readability of the 16 situational materials were evaluated, and the scores were scored from 1 to 5 (Jones et al., 2019; Yuan, 2022; Zhang, 2020; Zhao, 2022).

##### 2.1.2.1 Assessment of legality

Taking the legitimacy of the materials assessed by the participants as the dependent variable, the legal materials and illegal materials in the simulated fraud materials as the grouping variables, and performing the one-way ANOVA. The results show that The legitimacy scores of the subjects under different legitimacy materials were significantly different at the significance level of 0.001, *F*(1, 286) = 17.194, *p* < 0.001. Further comparison of their mean values shows that the legality score of legal materials is significantly higher than that of illegal materials. This shows that the subjects think that the legitimacy of legal materials is higher and the legitimacy of illegal materials is lower, indicating that the subjects can well distinguish legal materials from illegal materials, and the categories of legal materials and illegal materials in the compiled materials are effectively manipulated.

##### 2.1.2.2 Assessment of illegal situational materials

To ensure the balance of illegal materials, we performed one-way ANOVA on the legality, authenticity, logic of the text, content rationality, and readability for each group, using fraud type as the grouping variable.

One-way ANOVA was performed with authenticity as the dependent variable and fraud type as the grouping variable. The result shows that the effect of fraud type is not significant, *F*(1, 158) = 0.006, *p* = 0.941. Therefore, we can draw a conclusion that there is no significant difference in authenticity between loss-avoiding and profit-seeking illegal materials.

Taking text logic as the dependent variable and fraud type as the grouping variable for one-way ANOVA, the result shows that the effect of fraud type is not significant, *F*(1, 158) = 0.110, *p* = 0.740. As the result, it shows that there is no significant difference in text logic between loss-avoiding and profit-seeking illegal materials.

Taking the content rationality as the dependent variable and fraud type as the grouping variable and performing one-way ANOVA, the results show that the effect of fraud type is not significant with *F*(1, 158) = 1.350, *p* = 0.247. It shows that there is no significant difference in the content rationality between loss-avoiding and profit-seeking illegal materials.

Taking text readability as the dependent variable and fraud type as the grouping variable and performing one-way ANOVA, the result shows that the effect of fraud type is not significant, *F*(1, 158) = 0.065, *p* = 0.799. It can be seen that there is no significant difference in text readability between loss-avoiding and profit-seeking illegal materials.

##### 2.1.2.3 Assessment of legal situational materials

To ensure the balance of legal materials, we performed one-way ANOVA on the legality, authenticity, logic of the text, content rationality, and readability for each group, using fraud type as the grouping variable.

Taking authenticity as dependent variable and fraud type as grouping variable and carrying out one-way ANOVA, the result shows that the effect of fraud type is not significant with *F*(1, 142) = 2.668, *p* = 0.105. Therefore, it shows that there is no significant difference in authenticity between loss-avoiding and profit-seeking legal materials.

Taking text logic as the dependent variable and fraud type as the grouping variable for one-way ANOVA, the result shows that the effect of fraud type is not significant with *F*(1, 142) = 1.250, *p* = 0.266. We can draw that I there is no significant difference in text logic between loss-avoiding and profit-seeking legal materials.

Taking content rationality as the dependent variable and fraud type as the grouping variable for one-way ANOVA, and the result shows that the effect of fraud type was not significant, *F*(1, 142) = 2.526, *p* = 0.114. It shows that there is no significant difference in content rationality between loss-avoiding and profit-seeking legal materials.

Taking text readability as the dependent variable and fraud type as the grouping variable for one-way ANOVA, the result shows that the effect of fraud type is not significant with *F*(1, 142) = 2.616, *p* = 0.108. As the result, there is no significant difference in text readability between loss-avoiding and profit-seeking legal materials.

To sum up, self-compiled simulated fraud situation materials can be effectively distinguished as fraud situations. What’s more, there is no significant difference in authenticity, logic, content rationality and text readability among different fraud types of materials within the legal material and illegal material groups.

### 2.2 Methods

#### 2.2.1 Experimental design

Adopting two-factor mixed design with 2 (Time pressure: experimental group, control group) × 2 (Fraud type: loss-avoiding type, profit-seeking type), in which time pressure is an inter-subject variable and fraud type is an intra-subject variable. Taking the susceptibility to online fraud as the dependent variable, which is measured with the method posted by Jones et al. (2019): During the experiment, the subjects need to use a four-point scale to judge the legitimacy of the materials, and the evaluation between 1 and 2 is regarded as “legal and true,” while the evaluation between 3 and 4 is regarded as “illegal and fake.” Measure the performance of subjects with reference to signal detection theory (Guo, 2019), we first calculate the hit rate P (H) and the false report rate P (FA) respectively, and the Z scores of the hit rate and false report rate are calculated, respectively, through PZO conversion, that is, Z_Hit_ and Z_False reporting_. Next, according to the formula d′ = Z_Hit_ − Z_False_ reporting, we can calculate the discrimination index of each subject d′, as a measure of the subjects’ susceptibility to online fraud. As the result, the smaller the d′ is, the weaker the discrimination of fraudulent materials and the lower the sensitivity of the subjects will be, that is, in other words, the stronger the susceptibility to online fraud will be.

#### 2.2.2 Procedures

**Figure 1:**
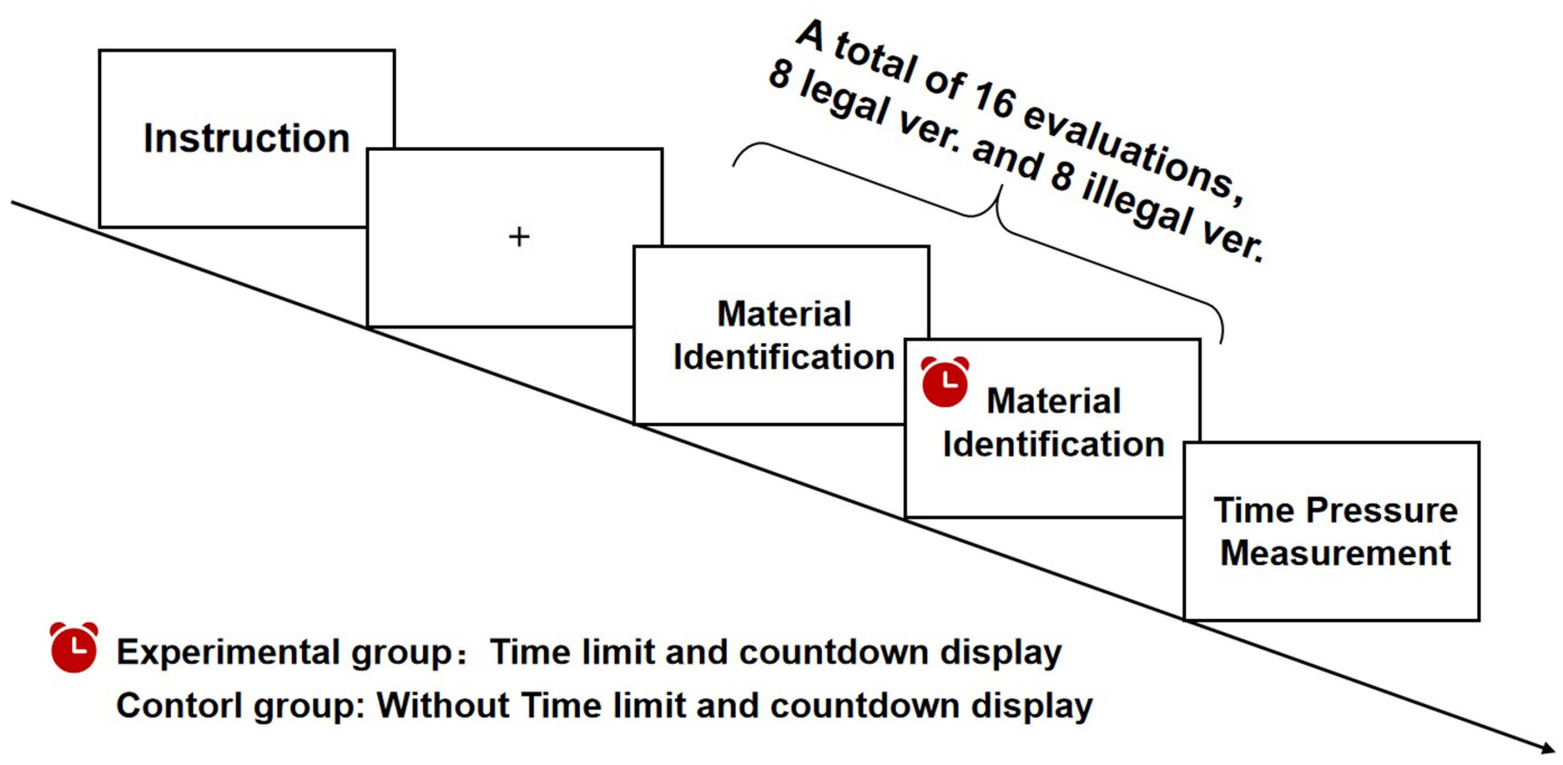
Experiment flow chart.

The experiment procedure is compiled in the data platform Credamo, which includes two stages: measurement stage and material legality evaluation stage. In the measurement stage, the two groups of subjects filled out the same scale, including basic demographic variables (gender, fraud experience, etc.), general interpersonal trust scale, thinking style scale and risk preference scale. After filling in the form, enter the material legality evaluation stage of the formal experiment. The instruction of the control group is: “Next, you will see a series of graphic and text situations. Please judge the legality of the situation according to your own experience (that is, the content For you: “1” = deceptive; “4” = credible) “The instruction of the experimental group is:” Next, you will see a series of graphic and text situations. Please judge the legitimacy of the situation based on your own experience (that is, the content is for you:“1” = deceptive;“4” = credible), there is a time limit for each page of text and question display. You need to complete the question within the specified time and click on the next page, otherwise the system will automatically jump to the next question. If the data is missing, it will affect You get the test fee normally, please pay attention to the time.” In the stage of material legitimacy evaluation, there is no time limit in the control group, while the time limit of each question in the experimental group is obtained by calculating the average answering time of the subjects in the control group. In order to ensure the most basic response time of the subjects, the answering time is rounded up (Bago et al., 2021; Bago and De Neys, 2017). For example, for the first question, the average answer time of the control group is 36,361 ms, then the answering time of the first question of the experimental group is limited to 40,000 ms. After the evaluation, the subjects completed the time pressure scale and tested the effectiveness of time pressure manipulation. The flow chart of this study is shown in Figure 1.

#### 2.2.3 Subjects

170 subjects were recruited on the Internet through WeChat group, with control group and experimental group 85 person respectively, of whom 83 were female. Wherein 86 people have suffered fraud to varying degrees. After the experiment, the subjects passed the logical polygraph questions will receive 5 yuan as the subject fee. Details of the subjects are shown in Table 2.

**Table 2:** Descriptive statistics of demographic variables.

Demographic variables	Group	Frequency	Percentage
Gender	Male	83	48.88%
	Female	87	51.12%
Monthly income	Under 1,000 yuan	8	4.71%
	1,500 yuan	44	25.88%
	2,000 yuan	52	30.58%
	2,500 yuan	23	13.54%
	More than 3,000 yuan	43	25.29%
Level of education	Junior high	1	0.60%
	High School/Vocational High School	2	1.20%
	Specialty	3	1.80%
	Undergraduate	150	88.15%
	Master	13	7.65%
	PhD	1	0.60%
Fraudulent experience	Yes	86	50.59%
	No	84	49.41%

#### 2.2.4 Data processing and analysis

Collect the material legality evaluation results of the subjects, and convert the scores according to the material properties. When the material is an illegal version, if the subject’s evaluation is 3–4 will be record 1 point, and no score will be obtained is the evaluation is 1–2, the score is the number of hits, through the formula P (H) = Yes/SN we can calculate the hit rate P (H). When the material is a legal version, if the subject’s evaluation is 3–4, the score should be rated as 1 point, and no score is obtained once it is evaluated as 1–2. The score is the number of false reports, and the false reported rate P (FA) can be calculated by the formula P (FA) = Yes/N. Re-pass PZO Conversion, calculating the Z Score of hit rate and false report rate, i.e., Z_Hit_ and Z_False reporting_. According to the formula d′ = Z_Hit_ − Z_False reporting_, we can calculate the discriminability index d′. Repeated measures ANOVA was performed on the data using SPSS 29.0, as well as an independent samples t-test for time pressure scores.

## 3 Results

### 3.1 Maneuverability test result

This experiment was compiled with Svenson, and Wang Dawei’s revised Time Stress Scale measures the time stress status of the participants (Wang, 2007). The total time pressure scores of the experimental group and the control group were independently sampled t Test, the results found that the time pressure score of the experimental group (*M* = 24.29, *SD* = 7.35) is significantly higher than the control group (*M* = 18.25, *SD* = 7.13), *t*(168) = 5.45, *p* < 0.001, *d* = 0.84. As the result, time pressure manipulation of the experiment works.

### 3.2 Difference test of subject variables

Before the formal experiment, this study measured the general interpersonal trust tendency, thinking style and risk preference of the subjects. The results showed that the general interpersonal trust score *F* = 0.55, *p* = 0.46, the style of thinking-rationality score *F* = 0.20, *p* = 0.65, thinking style-intuition score *F* = 2.60, *p* = 0.11, risk appetite score *F* = 1.53, *p* = 0.22. Therefore, there was no significant difference between the experimental group and the control group in the above dimensions.

### 3.3 Effect of time pressure and fraud types on online fraud susceptibility

**Figure 2:**
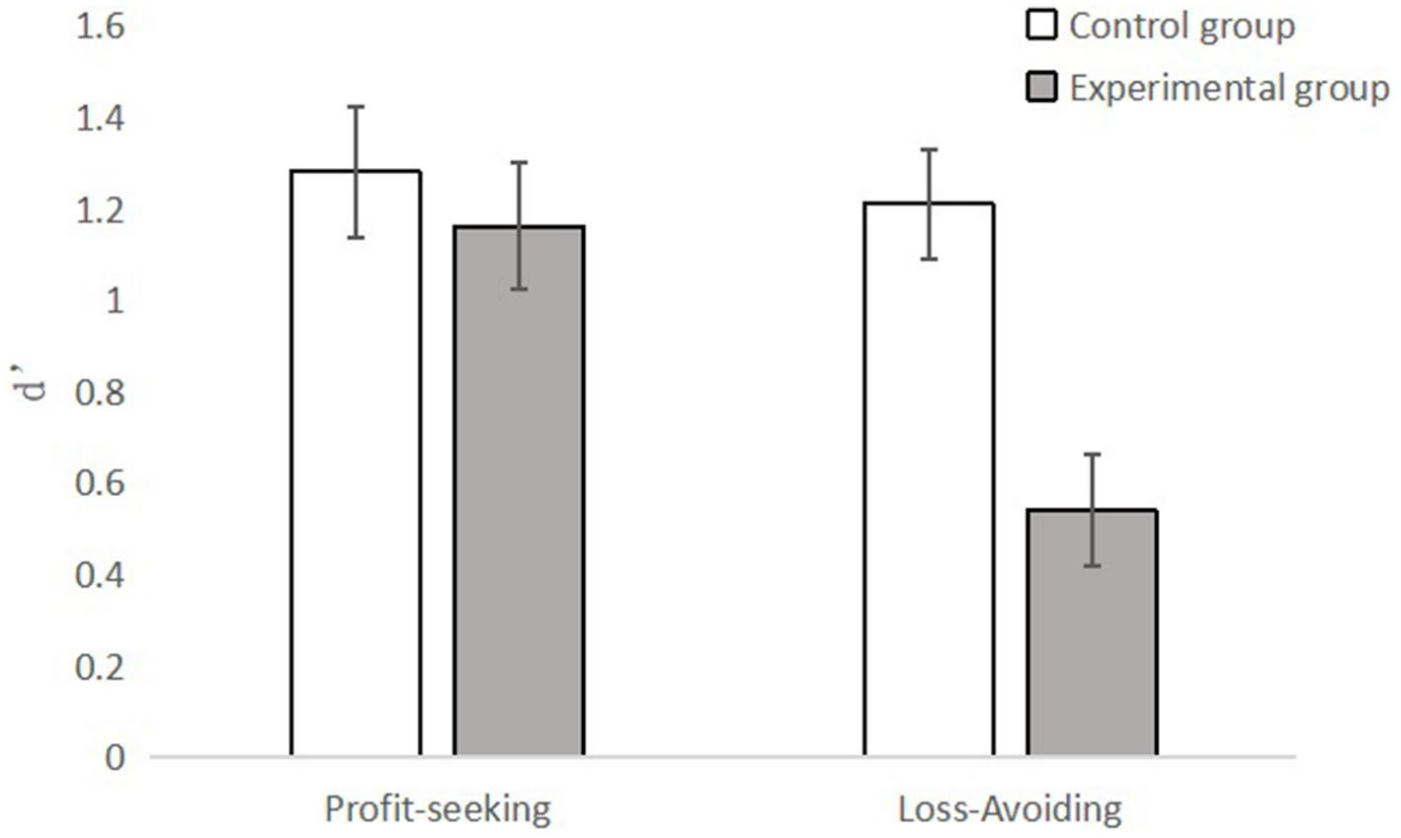
The interaction between time pressure and fraud type.

A repeated-measures ANOVA was performed on the participants’ discriminability index d′ for the legitimacy of the simulated fraud material, and the results are shown in Figure 2. The results showed that the main effect of time pressure was significant with *F*(1, 168) = 7.86, *p* = 0.006, *η*^2^ = 0.045, and the discriminability index of the experimental group d′ (*M* = 1.70, *SD* = 1.94) is significantly lower than the control group (*M* = 2.49, *SD* = 1.72). Therefore, the susceptibility to fraud of subjects under time pressure is significantly stronger than that of subjects without time pressure.

The main effect of fraud type is significant with *F*(1, 168) = 7.69, *p* = 0.006, *η*^2^ = 0.044. The discriminability index of loss-avoiding frauds d′ (*M* = 0.88, *SD* = 1.17) is significantly lower than profit-seeking frauds (*M* = 1.22, *SD* = 1.29). As the result, we can draw a conclusion that the susceptibility to fraud of the subjects under the loss-avoiding fraud is significantly stronger than that of the subjects under the profit-seeking fraud.

The interaction between time pressure and fraud type is obvious while *F*(1, 168) = 5.07, *p* = 0.026, *η*^2^ = 0.029. Further simple effect analysis shows that there is a significant difference between the susceptibility of online fraud between the experimental group and the control group for loss-avoiding frauds, that is, the existence of time pressure significantly enhances the susceptibility of online fraud of the subjects. For profit-seeking frauds, there is no significant difference between the fraud susceptibility of the experimental group and the control group, and the existence of time pressure does not significantly enhance the fraud susceptibility of the subjects. The results are shown in Figure 2.

## 4 Discussion

The study explores the effects of time pressure and fraud type on susceptibility to online fraud, and the experimental results verify all three hypotheses. In the test of time pressure perception after the experiment, the score of the experimental group was significantly higher than that of the control group, and the manipulation of time pressure in the experiment was effective.

### 4.1 Materials

A total of 16 simulated fraud situation materials are compiled in pre-study, and the content validity and various indicators of the materials are assessed. The assessment of the legitimacy of situational materials shows that compared with the materials of legal situations, the materials of illegal situations do have a lower legitimacy score in the legitimacy assessment. The results show that the self-compiled simulated fraud situation materials can effectively manipulate the legitimacy types of frauds. At the same time, for situational materials used for simulated fraud, there is no significant difference between loss-avoiding and profit-seeking materials in authenticity, text logic, content rationality and text readability, indicating that situational materials used in simulated fraud can well control additional variables to a certain extent. The simulated fraud situational material compiled in this study effectively manipulated and controlled the research variables, which can be used in subsequent experiments.

At the same time, the situational materials used in simulated fraud compiled in this study also provide materials for the research of fraud field for China research. Currently, psychological research on susceptibility mostly uses cyber attack testing and fraud material assessment (Gao, 2021). Among them, the network attack test focuses on foreign research relying on phishing emails, while the methods of online fraud in China are updated and iterated quickly. Consequently, it is difficult to targetly research on phishing emails to truly and comprehensively reflect the level of fraud susceptibility in line with the current situation in China. Therefore, most domestic scholars use the method of fraudulent material evaluation to study susceptibility. However, the fraud material evaluation methods used by most domestic scholars are adapted from the classic investment paradigm or only use a fixed self-made fraud situation, which makes the research on fraud susceptibility relatively limited, ignoring the role of fraud situation, means and types in the process of perceiving situational risk. The simulated fraud scenario materials compiled in this study are classified according to their legality and profit and loss framework, and each category uses four different means to create scenarios, which makes the scenarios more diverse, and it can improve the ecological validity. Therefore, it has a certain positive effect on the development of domestic fraud research.

However, due to the lack of interaction between the subjects and the experimental materials in the simulated fraud situation, it may not be possible to simulate the trust maintenance stage in the real fraud situation, and the explanatory power for the individual’s comprehensive fraud susceptibility is relatively limited. In the future, we can develop the simulated interaction process between fraudmers and victims, which can restore the real fraud situation as a whole as possible, making the experimental situation closer to the real fraud, and obtaining better external validity. At the same time, we can achieve better manipulation of variables by expanding the materials or further evaluating and classifying the materials.

### 4.2 General discussion

According to the experiment result, the result supports Hypothesis 1. The main effect of time pressure is significant, and under the condition of time pressure, the subjects’ discriminability of the legitimacy of simulated fraud situation is significantly lower than that without time pressure. In other words, the subjects’ susceptibility to online fraud under time pressure is significantly stronger than that without time pressure, which is similar to Luo’s experiment conclusion which claimed that the time pressure has a significant positive impact on the probability of college students being deceived (Luo et al., 2013).

Studies have shown that when victims are involved in fraud, fraudmers often induce victims to feel anxious and sleepy by applying time pressure and other methods, resulting in cognitive bias and emotional imbalance (Norris and Brookes, 2021), thereby enhancing the victim’s susceptibility to fraud. However, this study did not study the path by which time pressure affects fraud susceptibility. Therefore, it is suggested that further search for the intermediary and moderating variables between time pressure and fraud susceptibility can be carried out, and further clarify the mechanism of time pressure. Through laboratory simulation of fraud situations, Dong Hanchen explored the influence of cognitive load and time pressure on fraud decision-making (Dong and Qian, 2021). Among them, the main effect of time pressure is significant, and individuals are more willing to transfer money and are more susceptible to fraud under time pressure. From the perspective of different stages of fraud, this study focuses on the risk decision-making stage at the final transfer, while this study focuses on the fast trust stage, which shows that time pressure is quite effective in the whole process of fraud.

The experiment result supports the Hypothesis 2, the main effect of fraud type is significant. In the loss-avoiding fraud, the subjects’ discriminability of the legitimacy of the simulated fraud situation is significantly lower than that of the profit-seeking fraud, that is, the subjects’ fraud susceptibility under the loss-avoiding fraud is significantly stronger than that of the profit-seeking fraud. According to the prospect theory, the subjects under the return framework tend to adopt risk aversion strategies, while the loss framework tends to be risk preference. In this context, subjects in the loss-avoiding fraud situation may misjudge the risk information in the material, which in turn leads to. It is easy to trust fraudulent materials and show stronger susceptibility to online fraud. Loss-avoiding fraud causes negative emotions such as fear by creating “unexpected outcomes.” The fear-inducing content in fraudulent messages affects the process of information processing, which may lead to an increase in people’s acceptance of deceptive messages (Petty and Briñol, 2015). It can be seen that the results of this experiment are consistent with the proposed prospect theory advanced by Kahneman and Tversky. And it also reflects “Negative bias” proposed by Petty and Brinol.

This also reminds official departments and people who are susceptible to being deceived to pay special attention to emergency hedging frauds under loss-avoiding frauds. According to the prospect theory, individuals are more likely to have a preference for risks under loss-avoiding frauds, and then make misjudgments. Take the fraud disguised as the public security law as an example, this type of fraud often uses various reasons (for example, involving money laundering and illegal access) to convince the victim that he is about to be arrested by the local public security, and requires the victim to transfer the money to a “safe account” to prove his innocence. This is precisely to take advantage of the victim’s helplessness in the face of losses, weaken the victim’s ability to identify suspicious clues, and finally let the victim transfer money and fall into a trap.

The experimental result supports the Hypothesis 3, the interaction between time pressure and fraud type is significant. For loss-avoiding frauds, the existence of time pressure significantly enhances the subjects’ susceptibility to online fraud. For profit-seeking frauds, the existence of time pressure did not significantly enhance the subjects’ susceptibility to online fraud. This is consistent with the theories of previous related studies. Fraud theory shows that the ability to distinguish fraud situations lies not in deeper search, but in the intensity of fraud clues. According to deception theory, the identification of inconsistent cues (e.g., too-good-to-be-true guarantees, exaggerated claims of product merits) is necessary for successful detection (Grazioli, 2004). In profit-seeking frauds, “gaining benefits” is a strong inconsistent deceptive clue, so the subjects immediately make a “fraudulent” judgment. It can also be seen from the reaction time that the reaction time of loss-avoiding frauds is slightly higher than that of profit-seeking frauds. However, because the volume difference of materials, graphics and texts is not taken into account, and there are few sample situations, it is impossible to compare the statistical differences. Nevertheless, the results of deception theory can be reflected, since loss-avoiding frauds do not have the “strong fraud clues” in profit-seeking frauds, individuals need longer cognitive processing time and higher cognitive resources for loss-avoiding frauds, which requires more systematic processing. Compared with positive stimuli, negative stimuli are preferentially detected at lower exposure levels and elicit stronger or faster responses than positive events (Aarts and Dijksterhuis, 2003). However, individuals in loss-avoiding fraud situations receive more negative stimuli, and they tend to react immediately, and the delicate systematic processing process is blocked. Consequently, it is not conducive to the subjects’ identification and evaluation of fraud clues, which has a stronger impact on the subjects’ susceptibility to online fraud.

The results of this study prove that there is a significant interaction between time pressure and fraud types, but the mechanism behind the interaction has not been fully explored. According to equate-to-differentiate theory, time pressure is likely to affect the subjects’ feelings of the difference between the best outcome and the worst outcome in different profit and loss situations, and then affect the accuracy of the subjects’ judgment. It is suggested that subsequent research can focus on the mechanism behind the interaction and further explore its path and build a model.

The simulated fraud materials used in this study have high ecological validity, but in terms of method, the most suitable fraud situation is to use real cyber attacks to measure individuals’ susceptibility to online fraud. So far, only phishing email fraud has been studied by network attack. Although this method has certain ethical problems, after certain adjustments, it should become a more reasonable research method that can fit different fraud methods.

With the development of Internet technology, fraud technologies are constantly iterating and emerging in endlessly. For example, the development of AI face-changing technology makes it possible for fraudmers to steal other people’s facial information. As the result, even if video communication is used in the future, there is no guarantee that the person you communicate is who you think. But no matter how novel the fraud is, the logic and psychological mechanism behind it are similar. Therefore, this study can provide a basis for further psychological mechanism research and fraud intervention education in the future. In the process of fraud intervention education, by explaining the means and principles of fraud to citizens, as well as the common means in fraud (such as creating time pressure, creating accidents, unexpected gains, etc.), potential victims can better understand and internalize anti-fraud knowledge, enhance their ability and sensitivity to fraud situations, and more scientifically and effectively realize the education and protection of susceptible people from the source.

## Data Availability

The raw data supporting the conclusions of this article will be made available by the authors without undue reservation.

## References

[ref1] Aarts H. Dijksterhuis A. (2003). The silence of the library: environment, situational norm, and social behavior. J. Pers. Soc. Psychol. 84, 18–28. doi: 10.1037/0022-3514.84.1.18, PMID: 12518968

[ref2] Bago B. Bonnefon J. F. De Neys W. (2021). Intuition rather than deliberation determines selfish and prosocial choices. J. Exp. Psychol. Gen. 150, 1081–1094. doi: 10.1037/xge0000968, PMID: 33119351

[ref3] Bago B. De Neys W. (2017). Fast logic?: examining the time course assumption of dual process theory. Cognition 158, 90–109. doi: 10.1016/j.cognition.2016.10.014, PMID: 27816844

[ref4] Ben Zur H. Breznitz S. J. (1981). The effect of time pressure on risky choice behavior. Acta Psychol. 47, 89–104.

[ref5] Cacioppo J. T. Gardner W. L. Berntson G. G. (1997). Beyond bipolar conceptualizations and measures: the case of attitudes and evaluative space. Personal. Soc. Psychol. Rev. 1, 3–25. doi: 10.1207/s15327957pspr0101_2, PMID: 15647126

[ref6] Chen H. Zhao L. Guo S. Mo L. (2023). How do telecom frauds lead to false beliefs: influencing factors, explanatory theories, and research prospects. J. South China Normal Univ. (Soc. Sci. Ed.). 2.

[ref7] China Internet Network Information Center (2023). The 52nd statistical report on the development of the internet in China. Available online at: https://www.cnnic.net.cn/n4/2023/0828/c88-10829.html (Accessed October 26, 2023).

[ref8] Dong H. Qian X.. (2021). The influence of cognitive load and time pressure on fraud decision-making. Proceedings of the 23rd national psychological academic conference (Volume I).

[ref9] European Commission. (2020). Survey on “scams and fraud experienced by consumers”: final report. Available online at: https://ec.europa.eu/info/sites/info/files/aid:development_cooperation_fundamental_rights/ensuring_aid:effectiveness/documents/survey_on_scams_and_fraud_experienced_by_consumers_-_final_report.pdf (Accessed March 16, 2025).

[ref10] Gao Y. (2021). Influencing factors of susceptibility to online fraud among college students. [Master’s thesis]. Zhejiang, China: Zhejiang University.

[ref11] Gee J. Button M. (2019). The financial cost of fraud 2019. Available online at: http://www.crowe.ie/wp-content/uploads/2019/08/The-Financial-Cost-of-Fraud-2019.pdf

[ref12] Grazioli S. (2004). Where did they go wrong? An analysis of the failure of knowledgeable internet consumers to detect deception over the internet. Group Decis. Negot. 13, 149–172. doi: 10.1023/B:GRUP.0000021839.04093.5d

[ref13] Guo X. (2019). Experimental psychology. Beijing: People's Education Press, 248.

[ref14] Hanoch Y. Wood S. (2021). The scams among us: who falls prey and why. Curr. Dir. Psychol. Sci. 30, 260–266. doi: 10.1177/0963721421995489, PMID: 40115652

[ref15] Harrison B. Svetieva E. Vishwanath A. (2015). Individual processing of phishing emails: how attention and elaboration protect against phishing. Online Inf. Rev. 40, 265–281. doi: 10.1108/OIR-04-2015-0106

[ref16] Harrison B. Vishwanath A. Rao R. (2016). A user-centered approach to phishing susceptibility: the role of a suspicious personality in protecting against phishing. 49th Hawaii international conference on system sciences (HICSS), pp. 5628–5634.

[ref17] Hu W. Hu Y. (2009). The influence of time pressure on framing effects in risk decision-making. Psychol. Sci. 3, 694–696. doi: 10.16719/j.cnki.1671-6981.2009.03.016

[ref18] Johnson P. E. Grazioli S. Jamal K. (1993). Fraud detection: deception and intentionality in cognition. Account. Organ. Soc. 18, 467–488. doi: 10.1016/0361-3682(93)90042-5

[ref19] Johnson P. E. Grazioli S. Jamal K. Berryman G. (2001). Detecting deception: adversarial problem solving in a Low Base rate world. Cogn. Sci. 25, 355–392. doi: 10.1207/s15516709cog2503_2

[ref20] Johnson P. E. Grazioli S. Jamal K. Zualkerman I. A. (1992). Success and failure in expert reasoning. J. Organ. Behav. Hum. Decis. Process. 53, 173–203. doi: 10.1016/0749-5978(92)90061-B

[ref21] Jones H. S. Towse J. N. Race N. (2015). Susceptibility to email fraud. Int. J. Cyber Behav. Psychol. Learn. 5, 13–29. doi: 10.4018/ijcbpl.2015070102

[ref22] Jones H. S. Towse J. N. Race N. Harrison T. (2019). Email fraud: the search for psychological predictors of susceptibility. PLoS One 14:e0209684. doi: 10.1371/journal.pone.0209684, PMID: 30650114 PMC6334892

[ref23] Kahneman D. Tversky A. (1979). Prospect theory: an analysis of decision under risk. Econometrical 47, 263–291. doi: 10.2307/1914185

[ref24] Kim D. Kim J. (2013). Understanding persuasive elements in phishing e-mails: a categorical content and semantic network analysis. Online Inf. Rev. 37, 835–850. doi: 10.1108/OIR-03-2012-0037

[ref25] Li S. (1994). Equate-to-differentiate theory: a coherent bi-choice model across certainty, uncertainty and risk. Sydney, Australia: University of New South Wales.

[ref26] Li S. (2001). Equate-to-differentiate: the role of shared and unique features in the judgment process. Aust. J. Psychol. 53, 109–118. doi: 10.1080/00049530108255131, PMID: 40101104

[ref27] Li S. (2005). Choice reversal under certainty, uncertainty, and risk: an explanation of the "qi-dang-bie" choice method. Acta Psychol. Sin., 4, 427–433.

[ref28] Lindenberg S. (2001). “Social rationality versus rational egoism” in Handbook of sociological theory. ed. Turner J. (New York: Kluwer Academic/Plenum Publishers), 635–668.

[ref29] Liu Y. Sun Y. (2014). New perspectives on the study of framing effects in behavioral decision-making: from risk decision to intertemporal choice, from verbal frames to graphical frames. Adv. Psychol. Sci. 22:1205. doi: 10.3724/SP.J.1042.2014.01205

[ref30] Luo H. (2017). Research on telecom network fraud issues and governance strategies. [Master’s thesis]. Wuhan, China: Central China Normal University.

[ref31] Luo Y. (2022). The impact of neuroticism on individual susceptibility to deception under loss-based fraud conditions. [Master’s thesis]. Zhejiang, China: Zhejiang University.

[ref32] Luo X. Zhang W. Burd S. Seazzu A. (2013). Investigating phishing victimization with the heuristic–systematic model: a theoretical framework and an exploration. Comput. Secur. 38, 28–38. doi: 10.1016/j.cose.2012.12.003

[ref33] Modic D. Anderson R. Palomäki J. (2018). We will make you like our research: the development of a susceptibility-to-persuasion scale. PLoS One 13:e0194119. doi: 10.1371/journal.pone.0194119, PMID: 29543845 PMC5854354

[ref34] Modic D. Lea S. E. G. (2013). Fraud compliance and the psychology of persuasion. Rochester, New York, USA: Elsevier: Social Science Research Network.

[ref35] Moody G. D. Galletta D. F. Dunn B. K. (2017). Which phish get caught? An exploratory study of individuals' susceptibility to phishing. Eur. J. Inf. Syst. 26, 564–584. doi: 10.1057/s41303-017-0058-x

[ref36] Norris G. Brookes A. (2021). Personality, emotion and individual differences in response to online fraud. Personal. Individ. Differ. 169:109847. doi: 10.1016/j.paid.2020.109847, PMID: 40115879

[ref37] Ordóñez L. Benson L. (1997). Decisions under time pressure: how time constraint affects risky decision making. Organ. Behav. Hum. Decis. Process. 71, 121–140. doi: 10.1006/obhd.1997.2717

[ref38] Petty R. E. Briñol P. (2015). Emotion and persuasion: cognitive and meta-cognitive processes impact attitudes. Cognit. Emot. 29, 1–26. doi: 10.1080/02699931.2014.967183, PMID: 25302943

[ref39] Skidmore M. Goldstraw-White J. Gill M. (2020). Vulnerability as a driver of the police response to fraud. J. Criminol. Res. Policy Pract. 6, 49–64. doi: 10.1108/jcrpp-11-2019-0068

[ref40] Vishwanath A. Herath T. Chen R. Wang J. Rao H. R. (2011). Why do people get phished? Testing individual differences in phishing vulnerability within an integrated, information processing model. Decis. Support. Syst. 51, 576–586. doi: 10.1016/j.dss.2011.03.002

[ref41] Wang D. (2007). An experimental study of the time pressure effect in the decision making process. [Dissertation]. Shanghai, China: East China Normal University.

[ref42] Wang J. Herath T. Chen R. Vishwanath A. Rao H. R. (2012). Research article phishing susceptibility: an investigation into the processing of a targeted spear phishing email. Trans. Prof. Commun. 55, 345–362. doi: 10.1109/TPC.2012.2208392

[ref43] Wegener D. T. Petty R. E. (2001). “Understanding the effects of mood through the elaboration likelihood and flexible correction models” in Theories of mood and cognition: a user's guidebook. eds. Martin L. L. Clore G. L. (Hove, UK: Psychology Press), 177–210.

[ref44] Yuan J. (2022). Empathy training effectiveness research based on empathetic motivation and attention bias. Wuhan, China: Central China Normal University.

[ref45] Zhang Y. (2020). The effect of Chinese character frequency on orthographic neighborhood effects. [Master’s thesis]. Jinan, China: Shandong Normal University.

[ref46] Zhao J. (2022). Eye movement research on anticipatory processing in Chinese text reading. [Master’s thesis]. Jinan, China: Shandong Normal University.

